# Molecular typing and epidemiological investigation of clinical populations of *Pseudomonas aeruginosa* using an oligonucleotide-microarray

**DOI:** 10.1186/1471-2180-12-152

**Published:** 2012-07-27

**Authors:** Annalisa Ballarini, Giovanna Scalet, Malgorzata Kos, Nina Cramer, Lutz Wiehlmann, Olivier Jousson

**Affiliations:** 1 Centre for Integrative Biology, University of Trento, Trento, Italy; 2 Department of Pathology and Diagnostics, University of Verona, Verona, Italy; 3 Klinik für Pädiatrische Pneumologie, Allergologie und Neonatologie, Medizinische Hochschule Hannover, Hannover, Germany

## Abstract

**Background:**

*Pseudomonas aeruginosa* is an opportunistic pathogen which has the potential to become extremely harmful in the nosocomial environment, especially for cystic fibrosis (CF) patients, who are easily affected by chronic lung infections. For epidemiological purposes, discriminating *P.aeruginosa* isolates is a critical step, to define distribution of clones among hospital departments, to predict occurring microevolution events and to correlate clones to their source. A collection of 182 *P. aeruginosa* clinical strains isolated within Italian hospitals from patients with chronic infections, i.e. cystic fibrosis (CF) patients, and with acute infections were genotyped. Molecular typing was performed with the ArrayTube (AT) multimarker microarray (Alere Technologies GmbH, Jena, Germany), a cost-effective, time-saving and standardized method, which addresses genes from both the core and accessory *P.aeruginosa* genome. Pulsed-field gel electrophoresis (PFGE) and multilocus sequence typing (MLST) were employed as reference genotyping techniques to estimate the ArrayTube resolution power.

**Results:**

41 AT-genotypes were identified within our collection, among which 14 were novel and 27 had been previously described in publicly available AT-databases. Almost 30% of the genotypes belonged to a main cluster of clones. 4B9A, EC2A, 3C2A were mostly associated to CF-patients whereas F469, 2C1A, 6C22 to non CF. An investigation on co-infections events revealed that almost 40% of CF patients were colonized by more than one genotype, whereas less than 4% were observed in non CF patients. The presence of the *exoU* gene correlated with non-CF patients within the intensive care unit (ICU) whereas the pKLC102-like island appeared to be prevalent in the CF centre. The congruence between the ArrayTube typing and PFGE or MLST was 0.077 and 0.559 (Adjusted Rand coefficient), respectively.

AT typing of this Italian collection could be easily integrated with the global *P. aeruginosa* AT-typed population, uncovering that most AT-genotypes identified (> 80%) belonged to two large clonal clusters, and included 12 among the most abundant clones of the global population.

**Conclusions:**

The ArrayTube (AT) multimarker array represented a robust and portable alternative to reference techniques for performing *P. aeruginosa* molecular typing, and allowed us to draw conclusions especially suitable for epidemiologists on an Italian clinical collection from chronic and acute infections.

## Background

Molecular typing is an important tool in epidemiologic studies of infectious diseases, for identifying identical or closely related strains, sources of infection, and for detecting cross-transmissions in the nosocomial environment. Epidemiological outbreaks of bacterial infections are usually caused by initial exposure to a single etiologic agent. Therefore, the bacteria identified in the outbreak are often genetically identical or clonally related as a consequence of microevolutions events (usually point mutations) of an ancestor strain [1]. Molecular typing represents a tool to elucidate the genetic diversity underlying important phenotypic features such as host specificity, pathogenicity, antibiotic resistance and virulence [1]. Through molecular typing it is also possible to monitor the spread and the genetic diversity of nosocomial pathogens such as *Pseudomonas aeruginosa*. This bacterium represents a threat for its antibiotic resistance and its capability to cause a wide range of infections in human, including urinary tract, blood, and skin infections, but predominantly respiratory tract infections. This opportunistic pathogen plays a particularly detrimental role in cystic fibrosis (CF) patients, causing chronic respiratory infections leading to high infection rate and morbidity [2]. The genome complexity of *P. aeruginosa* is assumed to be the major reason for the adaptation skills of this bacterium to various environmental niches and its ability to cause a wide range of infections. Its large genome (5–7 Mb) includes core genes, necessary for survival, and a wide set of accessory genes conferring functional peculiarities to individual strains [3]. Such genomic variability derives from the extended capability of this species to acquire or discard genomic segments via horizontal gene transfer and recombination [3].

Several comprehensive molecular typing techniques for discriminating among *P. aeruginosa* strains have been developed, based either on DNA banding patterns (e.g. restriction fragment length polymorphism (RFLP) and pulsed-field gel electrophoresis (PFGE)), on DNA sequencing (e.g. multilocus sequence typing (MLST) and genome sequencing) or on DNA hybridization (DNA macro- and micro-arrays) [1].

PFGE typing is considered the “gold standard” DNA banding pattern-based method, being the most discriminative for hospital epidemiologists, who need to monitor the effectiveness of infection control measures [4]. The PFGE method, generating genome-wide DNA fingerprints with rare-cutter restriction enzymes, is also a cost-effective method. Nevertheless, it is extremely labor-intensive and lacks comparability between laboratories [1]. Nowadays, a viable PFGE pulsotype database for *P. aeruginosa* is not available, as a consequence of the unsuccessful efforts to standardize protocols worldwide.

After PFGE, MLST has become one of the most popular genotyping techniques [5]. The MLST is a sequencing-based method, which identifies SNPs as well as genomic rearrangements in six or seven conserved genes. Its significant advantage over PFGE typing is to be high-throughput and highly reproducible, allowing reliable data comparison to public global databases. However, to date it is still an expensive method and it bears the *in silico* complexity associated to sequencing output. Overall, both DNA-banding pattern-based and sequencing-based methods present drawbacks, showing either low reproducibility (PFGE) or high realization costs (MLST).

DNA hybridization-based methods have recently become a promising alternative for high-throughput investigation of genetic markers defining bacterial genetic diversity and relatedness [1,6]. DNA macro- and micro-arrays methods represent in fact the optimal compromise between the cost-effectiveness of DNA banding pattern-based methods and the reproducibility of sequencing-based methods.

For *P. aeruginosa* typing, a species-specific oligonucleotide-microarray has been developed by Wiehlmann and collaborators [7]. This microarray is based on the ArrayTube (AT) platform (Alere-Technologies GmbH, Jena, Germany) and allows the genotyping of *P. aeruginosa* strains using 13 informative single nucleotide polymorphisms (SNPs) at conserved loci, the *fliCa/fliCb* multiallelic locus and the presence or absence of the *exoS/exoU* marker gene. [7]. These reference alleles are based on the *P. aeruginosa* PAO1 chromosome and are described to be informative with a frequency of > =15% for the rarer allele in the *P. aeruginosa* population [8].

In contrast to PFGE-based fingerprinting, the discrimination between isolates based on PAO1- and non PAO1-like alleles represent a limit for performing phylogenetic analyses since these alleles are based on few core genome loci subjected to diversifying selection and mutation rate is not fast enough to investigate evolutionary relationships. Similarly to MLST, which is based on housekeeping genes with high sequence conservation, the PAO1-based AT profiles are sufficiently stable over time to make the AT approach ideal for defining relatedness of isolates for epidemiologic purposes. In order to define the relatedness between genotypes, the eBURST algorithm can be applied [9], which divides bacterial populations into cluster of clones and potentially identifies the ancestral strain. This clustering algorithm is commonly applied to MLST data [9], but it is suitable to any typing method based on defined genetic elements [7,10,11].

Unlike MLST, which scans only genetic informative traits of the core genome, the AT multimarker microarray also analyzes the composition of the accessory genome through a set of 38 genetic markers, so defining the intra-clonal diversity and epidemiological gene pattern [7]. Moreover, the AT typing, as the MLST, produces a robust and informative genotyping identifying isolates to the strain level and allowing easy and reliable data comparison between laboratories worldwide [12]. The ArrayTube has been already employed for molecular typing of *P. aeruginosa* populations isolated from various environments [13-17] and it has been shown to be adequate even when other typing techniques produced inconsistent results [18].

This work reports the molecular typing of an Italian *P. aeruginosa* clinical collection (n = 182), performed with the AT microarray, and the investigation on the virulence genes/gene islands correlating to the strain source (infection type or location). Data from a set of strains were compared with the PFGE and MLST methods, focusing on the adequateness for epidemiological studies. The prevalence of specific virulence genes from the accessory genome in the identified cluster of clones was defined. AT data of our local population on independent isolates (n = 124) were clustered according to their genetic similarity and analyzed together with publicly available *P. aeruginosa* worldwide AT datasets.

## Results and discussion

### Genotyping by ArrayTube and comparison to reference typing techniques

182 *P. aeruginosa* isolates were collected from three Italian hospitals, located in Rovereto, Trento, and Verona. All strains were typed with the ArrayTube (AT), a DNA-based multimarker microarray. The AT array design and array experiments are available in the ArrayExpress database (http://www.ebi.ac.uk/arrayexpress) under accession numbers E_MTAB_1108 and A-MEXP-2179, respectively. Excluding all isolates with identical AT-profile collected from individual patients, although from different body sites, 124 independent-strains could be selected. Besides AT-typing, PFGE and MLST were performed on up to 105 strains of our collection, for comparison purposes. The AT-genotypes and virulence markers profiles, PFGE-clone types and MLST genotypes are provided as supplementary material (Additional file 1).

Concerning the AT-dataset, the AT-genotype was derived from the 13 SNPs markers plus the *fliCa/b* multiallelic locus and the *exoS/exoU* markers, as described by Wielhmann and collaborators [7]. Isolates with identical AT-genotype (i.e. identical hexadecimal code) and also identical pattern of AT virulence markers were defined as AT-clones, since they are genetically indistinguishable according to the AT approach. Isolates with identical AT-genotype but different pattern of virulence markers were referred to as isolates belonging to the same AT-clonal complex. Finally, isolates with different AT-genotypes but related, according to eBURST analysis, were defined as isolates belonging to the same AT-cluster of clones [7].

The AT-genotyping analysis revealed that the 182 collected strains belonged to 41 different AT-genotypes. The relative low genomic variation observed in strain-specific regions within the core genome was concordant with the high genetic conservation previously found by genomic sequencing for *P. aeruginosa* strains [19]. Each clonal complex, i.e. group of isolates with identical AT-genotype, comprised 3.0 +/− 5.1 isolates.

A set of strains of our collection was analyzed also with two genotyping techniques commonly used in microbiology, which are renowned as high resolution reference methods, i.e. pulsed-field gel electrophoresis (PFGE) and multi-locus sequence typing (MLST) [1]. Comparison with these techniques was performed to gain insights into differences/similarities between approaches and to verify results of previous research groups underlining the feasibility of the AT approach for epidemic strains [18].

The PFGE/*Spe*I typing was performed on 105 independent strains of our collection, and resolved 77 different fingerprints, defined as different PFGE clones or pulsotypes (Additional file 2), against the 32 AT-genotypes identified by microarray typing within the same set of isolates. Only 24.0% PFGE/*Spe*I clones appeared to be clonal complexes, according to the phylogenetic analysis, whereas AT-typing identified 15 multi-isolates AT-genotypes out of 32 (42.9%).

The determined Simpson’s diversity index (DI) Simpson’s index of diversitywas higher for PFGE than AT (DI = 0.992 for PFGE (0.989–0.996 95% CI); DI = 0.91 for AT (0.872–0.947 95% CI) and the global congruence between the typing methods was low (adjusted Rand coefficient = 0.077 (0.012–0.140 95% CI)).

The displayed greater discriminatory power of the PFGE technique compared to AT-typing was concordant with published data [18] and it is a consequence of the different definition of a clone on which these two techniques are based. PFGE/*Spe*I typing resolves isolates by their *Spe*I macrorestriction pattern, thus focusing on presence or absence of recognition sites for the selected genome-wide rare-cutter restriction endonuclease (around 36 *Spe*I sites on the reference *P. aeruginosa* PAO1 genome [20]). Differently, the ArrayTube genotyping is based on the knowledge of *P. aeruginosa* genome organization, and it recognizes presence or absence of 15 *a priori* well-known SNPs or gene markers (13 single nucleotide polymorphisms (SNPs) and 2 marker genes) [7]. Being the AT-markers less numerous than *Spe*I restriction sites and based solely on the PAO1-genome, they do not allow to perform phylogenetic analyses. However, they are well suitable for epidemiological studies, since they are not affected by the genome instability exhibited by some epidemic strains, which bias the discrimination power when routine methods are used [18]. For example, the isolates with genotype 4B9A, mostly found in CF patients, were dispersed in 4 different PFGE clones (D, MM, QQ and UU) (see Additional file 3). Another example is represented by genotype 6C22, comprising isolates from the same hospital (Verona) and even department (Hematology). According to the PFGE typing, they belonged to 2 different clones, HH and II although closely related (see Additional file 1). This example shows how the microarray typing, despite being less discriminative than PFGE provides a genotype definition which is particularly suitable for epidemiological studies. This finding is striking looking at isolates from the same patient. For example, 2 isolates from patient P54, presenting genotype 1BAE and identical virulence profile, were defined as the same epidemiological clone according to AT approach, but showed 2 different PFGE fingerprints.

Besides the evaluation of the discriminatory power of AT versus PFGE typing, we tested whether there was concordance between clusters of clones defined by those techniques. Out of 4 AT clusters of clones identified, only the 3 small clusters had the at least 50% of the clones defined as part of the same cluster by both AT and PFGE (see Additional file 3). The isolates from the main AT cluster instead (including 66 isolates from 11 AT-genotypes) were spread over 19 different PFGE pulsotypes.

MLST was also applied to a set of independent isolates (n = 80). Among them, 46 showed a known sequence type (ST) profile and were grouped in 16 MLST STs with 1 to 11 strains (see Additional file 1). The remaining 34 isolates were defined as new by the MLST Public Database (pubmlst.org/paeruginosa) (see Additional file 4). However, these 34 new MLST-profiles included 4 profiles deriving from a combination of known alleles not described in the public database, 10 profiles due to the presence of at least one novel allele, and the remaining 20 profiles with medium-quality sequence within one or multiple alleles, for which the allele type could not be univocally determined. Excluding the two isolates with > 2 alleles with medium-quality sequences, overall 48 MLST STs could be identified among the remaining 78 isolates, the majority of which were single-isolate ST groups (81.3%). The AT-approach identified within the same set of isolates a smaller number of AT-genotypes, precisely 24, more than half of which (54.2%) with multiple isolates (see Additional file 1).

This data suggested a higher discriminatory power of MLST in comparison to AT-typing, which could be explained by the much higher information content of sequence data on the 7 MLST-marker genes versus presence/absence of polymorphisms in single nucleotides within the 13 ArrayTube SNPs-markers. The Simpson’s index of diversity (DI), calculated on all 78 isolates, was indeed higher for MSLT than AT microarray typing (DI = 0.966 for MLST (0.946–0.987 95% CI); DI = 0.924 for AT (0.894–0.954 95% CI)), indicating a higher discriminatory ability of MLSTversus AT. However, the difference in discrimination ability was lower than for PFGE versus AT. Also, the global congruence between MLST and AT (adjusted Rand coefficient = 0.559 (95% CI)) was higher than for PFGE versus AT.

**Figure 1:**
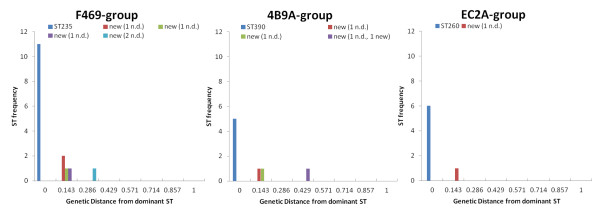
**Genetic distances between MLST DNA sequences within AT-groups.** The genetic distance between MLST DNA sequences is shown for AT-groups F469, 4B9A and EC2A. Each graph represents on the horizontal axis the genetic distance to the dominant MLST-ST within the AT-group and, on the vertical axis, the absolute frequency of each ST.

Focusing on the 3 AT-groups with the most MLST-typed isolates, i.e. F469, 4B9A and EC2A, we observed that within each of these groups, more than 62% of the isolates (68.8% for the F469 group, 62.5% for 4B9A and 75.0% for EC2A) had an identical MLST-profile, whereas the other isolates differed for 1 to 3 MLST-alleles from the dominant clone of the group. By computing the genetic distances between the MLST DNA sequences of the three AT-types, we observed that highest genetic distance was equivalent to 0.286 (isolate VRPS110) within the F469-group, 0.429 (isolate VRPS97) in the 4B9A-group and 0.143 (isolate FC17) within the EC2A-group (see Figure 1).

Looking at the three larger AT-groups, the exclusion of all isolates with medium-quality allele sequences increased the number of isolates with identical MLST ST within each group. In detail, all 11 isolates from the F469 group had an identical MLST-profile, i.e. ST214, all 6 isolates from EC2A had MLST ST260 and 5 out of 6 isolates within the 4B9A-group corresponded to a unique sequence type, i.e. ST390.

We observed that excluding all isolates with one or more medium-quality allele sequences, the disagreement between the two techniques further decreased, as shown by the similarly high Simpson’s index of diversity and the higher global congruence between methods calculated on the 53 isolates with good quality allele sequences (DI = 0.926 for MLST (0.888–0.964 95% CI); DI = 0.922 for AT (0.886–0.959 95% CI); adjust Rand coefficient = 0.912 (95% CI)).

Overall, the AT-approach was comparably informative to MLST for genotype definition and additionally provided information on the accessory genome. Thus, we employed the AT multimarker microarray to define genotype and virulence profile for all strains of our collection, identify potential correlations between strain source and AT-genotype or virulence gene pattern, and relate our data to the global AT population.

### Correlation between AT-genotype and strain source

The strains were collected from three hospitals and were isolated from patients affected by one of these two different infection-types: chronic infections (from CF patients) and acute infections (from patients in the intensive care unit (ICU) or other hospital departments (OTHER)). To investigate whether strain AT-genotype correlated with strain source, we grouped the 124-independent isolates of our collection according to their AT-type, infection type or hospital location.

**Figure 2:**
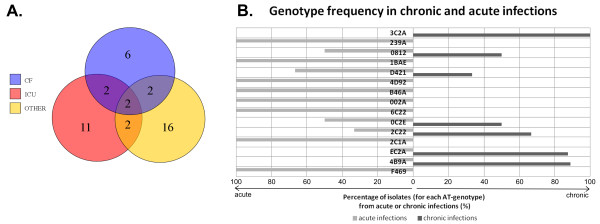
**Distribution of AT-genotypes among chronic and acute infections. A**. Venn’s diagram of the 41 AT-genotypes among chronic and acute (ICU and OTHER) infections. **B**. Histogram plot of frequency data percentages for the 15 multi-isolate AT-genotypes identified. Distributions were calculated from the 124 independent *P. aeruginosa* isolates of our collection.

Overall, 33 out of 41 AT-genotypes were exclusively found in either CF or non-CF (ICU, OTHER) and, among the multi-isolate clones, 11 out of 15 AT-types showed to be prevalent (with more than 80% isolates each) in either chronic or acute infections (see Figure 2), supporting previous evidence of an association of clones to a particular source [15]. The existence of infection-type specific clones is still under debate [12,21] and the reduced size of some of our clonal complexes did not allow us to draw statistically significant conclusions on the overall behaviour but rather to gather information on individual genotypes.

Among the 15 multi-isolates AT-genotypes of our collection 4B9A, EC2A, 3C2A were more frequently (more than 80% of their isolates) associated to chronic infections, whereas F469, 2C1A, 6C22 to acute infections (see Figure 2). Despite the unbalanced distribution of isolates from chronic and acute infections in our settings depending from the hospital location (Additional file 5), we assumed that a similar distribution of clones would be observed in the three hospitals, given the short geographical distance between their locations. Thus, we attributed the observed prevalence of specific AT-genotypes exclusively to the infection type. Also, some of the individually identified correlations confirmed findings of other research groups [7].

**Figure 3:**
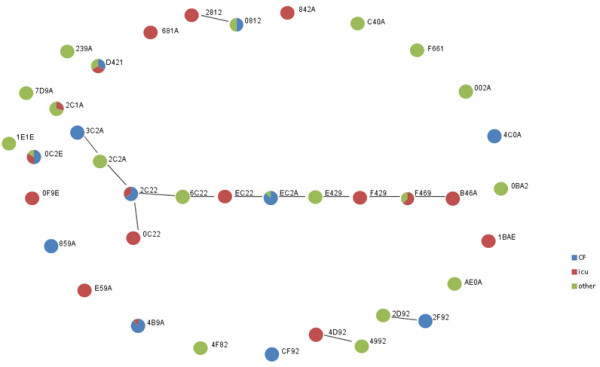
**Cluster of AT-clones identified within the 124 independent isolates of our*P.aeruginosa*collection.** Cluster of clones were identified by using the eBURST algorithm performed on 13 SNPs plus the multiallelic ***fliCa/fliCb*** gene. The colour code indicates for each genotype the % of isolates associated to CF patients, patients from the intensive care unit (ICU) or other hospital units (OTHERS). Novel clones (not described in other studies) are highlighted by a rectangular box.

AT-genotypes were clustered according to their genetic similarity, using the eBURST algorithm on the 14 AT-markers designed for genotyping (13 SNPs and *fliCa/fliCb*) [15]. By excluding the *exoU* and *exoS* markers, 3 clones collapsed into others, precisely clones F468 into F469 and EC28, EC29 into EC2A (see Figure 3).

Focusing on chronic associated isolates, the 4B9A AT-genotype belonged to the largest AT clonal complex and correlated to chronic infections, being 88.9% of its isolates collected from CF patients (see Figure 2), in contradiction with other collections in which this AT-genotype was described within keratitis, environmental and COPD samples [14,15,17]. As for the 4B9A AT-genotype, EC2A, known as CHA strain [7], was also mostly associated to the CF patient cohort (see Figure 2). The identified correlation is supported by previous studies and the mechanism of action of strains with this AT-genotype on human blood cells has been already elucidated [22]. 3C2A was exclusively CF-associated, but it has been previously described as a frequent AT-type both in CF and non-CF patients [7]. Among the multi-isolate AT-genotypes, only one novel one(i.e. 0C2E) out of 3 novel genotypes was identified also in CF patients, although in 50% of the cases only.

**Figure 4:**
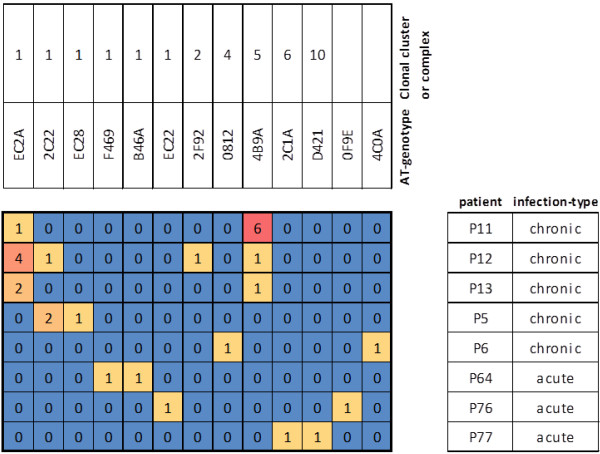
**Patients co-infected by isolates belonging to 2 or more AT-genotypes.** Patients with chronic or acute infections infected by isolates with different AT-genotypes are shown. Above each AT-genotype, the corresponding clonal cluster ID or clonal complex ID is indicated (see Table S1). The number of independent isolates identified for each genotype is indicated in squares and highlighted by a colour code.

An investigation on co-infections events, taking in account the 124-independent isolates collection, revealed that almost 40% of our CF patients were colonized by more than one AT-genotype, among which the most frequent were again 4B9A and EC2A but also the 2C22 AT-types (see Figure 4). Interestingly, isolates typed as 4B9A and EC2A, when present, were always co-colonizers (i.e. patients P11, P12, P13). According to the eBURST analysis shown in Figure 3, these two AT-genotypes showed low SNP-profile similarity and were classified as unrelated by the eBURST analysis of our collection being part of different cluster of clones. Looking at the accessory-genome markers, the isolates with 4B9A and EC2A AT-type presented an identical pattern of virulence genes/gene islands (see Additional file 1). Among the 5 patients infected by more than one AT-genotype, only an individual patient (P12) was co-infected by two strains from the same cluster of clones, with EC2A and 2C22 AT-genotype. Despite the similar SNP-pattern characterizing these two strains, they differ in the set of virulence markers for the presence of the fla-glycosylation island (detected in EC2A but not in 2 C22). Another patient (P5) may be infected by two highly similar strains, being typed as EC28 and 2C22. Excluding the *exoS/exoU* AT core-genome marker, the EC28 isolate was in fact genotypically identical to the EC2A one, thus becoming part of the cluster of clone 1, together with the co-infecting 2C22 strain.

As for chronic infections, acute infections were also found to be dominated by specific AT-genotypes. In particular, F469, the absolute most frequent AT-type within our collection, was exclusively associated to acute infection (see Figure 2). F469 isolates were primarily found (62.5%) in patients from the intensive care unit (ICU), carrying severe acute infections, and secondly (37.5%) in patients from the hematology unit (OTHER), affected as well by acute infections (see Additional file 1). The correlation between F469 and acute infections is well supported by other AT studies, identifying this AT-genotype within environmental samples and keratitis patients [15,17] (see Table 1).

**Table 1:** **The*Pseudomonas aeruginosa*AT-genotypes identified in our study and their presence in publicly accessible AT-databases**

**AT genotypes**	**Presence in other databases (reference)**
0812, 239A, 2C1A, 3C2A, C40A, D421, E429, F429	[7,14,15,17]
F469	[7,15,17]
F661	[7,14,17]
4B9A	[12,15,17]
2F92	[7,15]
1BAE	[7,14]
0C2E, 6C22, EC22, EC29, EC2A	[7,17]
2C22	[14,17]
0F9E, 4992, 7D9A, E59A	[7]
002A, 0BA2, 2C2A, CF92	[17]
0C22, 1E1E, 2812, 2D92, 4C0A, 4D92, 4F82, 681A, 842A, 859A, AE0A, B46A, EC28, F468	none

The AT-genotypes identified in our study were search in other published AT-databases [7,14,15,17]. Genotypes were grouped according to the AT-databases sharing them.

The 2C1A AT-genotype, better known as Midlands 1 [23], was also exclusively identified in acute infection and predominantly (71.0%) in patients affected by an acute infection of the respiratory apparatus (see Additional file 1). Our finding is in contrast with previous data, describing the Midlands 1 as the second most common clone in CF centres in Great Britain [23]. The 6C22 AT-type was exclusively isolated from blood infections in Verona, and it has been previously mainly reported as environmental [7,17]. Besides known AT-genotypes, 2 novel ones, B46A and 4D92, were identified. Multiple isolates belonged to these AT-types which were exclusively found in patients affected by acute infections, precisely within the intensive care unit (ICU) of the hospital located in Verona.

Few co-infection events (less than 4%) could be observed in patients with acute infections, in comparison to those observed in patients affected by chronic infections (almost 40%) (see Figure 4). Moreover, the co-infecting strains differed in their AT-type in each patient and, according to the eBURST analysis of our collection, only one patient (P64) was co-colonized by two strains with AT-genotypes belonging to the same cluster of clones (i.e. F469 and B46A). B46A showed a different set of virulence genes and gene islands than F469, precisely for the absence of *exoU* and the presence of the PAPI1-island.

### Correlation between genes or gene islands of the accessory genome and strain source

**Figure 5:**
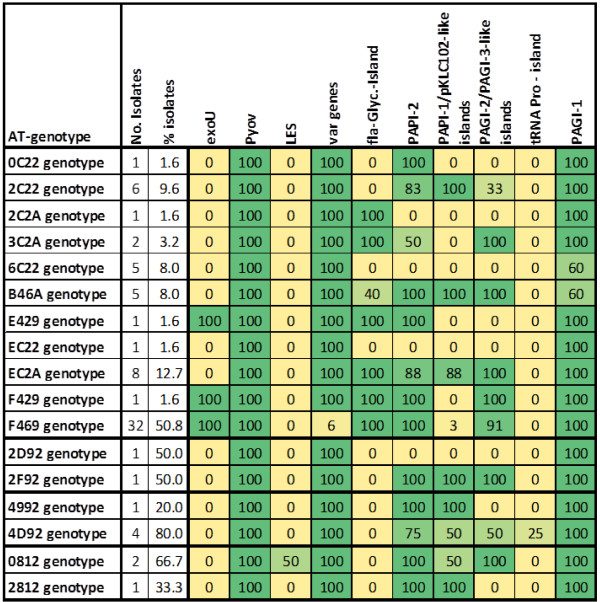
**Identification of the prevalent genes/gene islands from the accessory genome for each AT-genotype belonging to a cluster of clones in our collection.** The frequency of each gene/gene island is shown within each square as a percentage of isolates within each AT-genotype and highlighted by a colour code. The frequency data and number of isolates refers exclusively to independent isolates.

The ArrayTube multimarker microarray allowed not only discriminating among *P. aeruginosa* genotypes with proper resolution for epidemiological investigations, but also defining a molecular profile of key accessory genes and gene islands and their correlation to infection type or department. The prevalence of each accessory genome marker was determined among AT-genotypes belonging to the 4 cluster of clones identified by eBURST analysis in our collection of independent isolates (n = 124) (see Figure 5). The main cluster of clones within our strain collection (cluster 1) was characterized by genes and gene islands shared by all AT-genotypes of the cluster (e.g. the *fpvA* gene encoding the pyoverdine outer membrane transporter), but also by AT-type specific genomic regions such as the *exoU* gene, the LES-specific mutations or the fla-glycosilation island.

A statistical analysis [24] revealed that the presence of the *exoU* gene positively correlated (p < 0.01) with the ICU department, which hosted patients with severe acute infections. This finding was concordant with the known function of the protein encoded by the *exoU* gene, a potent cytotoxin causing damages in lung tissue, thus not compatible with chronic infections [25]. On the contrary, the *exoS* gene, described as mutually exclusive with the *exoU* gene [26], was associated in this study to CF strains (p < 0.01).

Besides the *exoU* gene, a positive correlation was also identified between the genes belonging to the pKLC102-like island, in particular genes encoding for pKL-1, pKL-3, pKLC adhesion, pKLC fatty acid synthase (all with p < 0.01), the pKLC conserved hypothetical protein (with p < 0.05) and the infection-type (CF or non-CF). These 5 genes were prevalent in CF strains, not only in our strain collection but also in the global population (p < 0.01, except for pKL-3, with p < 0.05) [7] and COPD isolates (p < 0.01, except for pKL-1, with p < 0.05, and the pKLC conserved hypothetical protein, which does not show a statistically significant correlation) [14]. The function of most of the genes belonging to this island has not been deciphered yet, but it is known that the PAPI-1/pKLC102-like members encode virulence factors, such as cytotoxins, pili, fimbriae and regulators of biofilm synthesis and antibiotic resistance [27]. Given the known functions of this island, the identified positive correlation to chronic infections was unexpected, as it has been demonstrated that *P. aeruginosa* reduces its acute virulence during the adaptation to the CF lung environment [28]. Nevertheless, Rakhimova and collaborators [14] showed that the pKL-3 gene was associated to a prolonged colonization time in a minority of *P. aeruginosa* strains in COPD patients [14], whose lung colonization pattern by *Pseudomonas* strains is comparable to the one observed in CF patients.

### Analysis of the AT-genotypes identified within the publicly available population studies

An intrinsic feature of the AT technology is to be standardized and therefore to guarantee reliable data comparison between genotyping studies performed worldwide in different laboratories [7]. In order to gain further information on the 124-independent strains of our collection, we compared them with a global database, obtained by retrieving information from 4 publicly available AT-datasets, comprising a total of 698 isolates [7,14,15,17]. These datasets comprised 240 strains of diverse habitat and geographic origin [7], 134 strains collected from patients affected by chronic obstructive pulmonary disease [14], 63 strains isolated from keratitis [15], and 381 environmental isolates from rivers [17].

Our 124-independent strain collection included 27 genotypes previously described [7,14,15,17] and 14 which have never been previously reported (see Table 1). Among the 27 already described AT-genotypes, it is interesting to notice that 8 of them (D421, 3C2A, C40A, 2C1A, 239A, 0812, E429 and F429) were shared by all collections [7,14,15,17] and were all among the 16 most abundant in the global *P. aeruginosa* population [7].

An eBURST analysis using 15 markers (13 SNPs, the multiallelic *fliCa/fliCb* locus and *exoS/exoU*) was performed to illustrate the similarities between SNP profiles of our and other collections, typed by the AT method. As shown in Additional file 6, the eBURST analysis revealed the presence of 2 main clusters of clones and 3 small ones (with 2–3 genotypes each). Most AT clones also previously described (25 out of 27) belonged to the 2 large clusters, 12 of which were among the 16 most abundant clones in the global *P. aeruginosa* population, namely D421, F469, 1BAE, 2C1A, 0C2E, 239A, 0812, C40A, E429, EC29, F429 and 3C2A [7]. All novel AT clones except one (1E1E) were part of the 2 large clusters or gave rise to a small cluster including a previously described strain (i.e. 4F8A) (see Additional file 6, clones enclosed in rectangular frames).

Overall, 84.2% clones of the local population (32 out of 38) were equally divided into the two large clusters of clones and almost 30% (11 out of 38) were primary founders, i.e. E469, E429, D421, F429, C40A, EC2A, 0C2E, 0812, 2C1A, 239A, and 1BAE (see Additional file 6, underlined clones). Among the 11 primary founders identified within our collection, 5 were known to be abundant clones in the global *P. aeruginosa* population [7], confirming their dominant role in the global *P. aeruginosa* population.

## Conclusions

The ArrayTube multimarker-microarray represented a reliable and reproducible tool for *P. aeruginosa* molecular typing. Genotypic data was readily comparable to public databases and allowed to draw conclusions on the correlation between isolates and infection type or department. A comparison with reference genotyping techniques showed how the AT provides a genotypic profile which is not biased by genome variations within unknown or not informative regions, and defines additionally epidemiological features to identifying the causative strain and transmission pattern in epidemiological outbreaks.

## Methods

### Strain collection

The *P. aeruginosa* strain collection (see Additional file 1) consisted of 107 isolates from the “Borgo Roma” Hospital (Verona, Italy), 14 from the “Santa Chiara” Hospital (Trento, Italy) and 61 cystic fibrosis isolates from the “Santa Maria del Carmine” Hospital (Rovereto, Italy). Strains were confirmed as *Pseudomonas aeruginosa* isolates using the biochemical assay API-20NE gallery (Biomerieux, Inc., Durham, NC), according to the manufacturer’s instructions. Results were further confirmed by PCR amplification of the *ecfX* gene, as previously described [29].

All information on the 182 isolates, their clinical source and their complete AT-profiles is available in the ArrayExpress database (http://www.ebi.ac.uk/arrayexpress) under accession number E_MTAB_1108.

### ArrayTube (AT) microarray platform

Each oligonucleotide-microarray for *P. aeruginos*a typing was located at the bottom of the ArrayTube (AT), purchased at Alere Technologies GmbH (Jena, Germany). The core genome was represented by 13 single-nucleotide polymorphisms (SNPs), the multiallelic *fliCa/b* locus and the *exoU*/*exoS* genes, while the accessory genome was represented by 38 genetic markers [7]. The array design is provided in the ArrayExpress database (http://www.ebi.ac.uk/arrayexpress) [30] under accession number A-MEXP-2179.

### Multimarker microarray typing protocol

DNA labeling and amplification were performed on *P. aeruginosa* colony DNA by linear amplification in the presence of dTTP: biotin-16-dUTP as suggested by the manufacturer (Alere Technologies GmbH, Jena, Germany). Hybridization was detected by colorimetry, using a streptavidin-horseradish peroxidase (HRP) conjugate and a HRP substrate, according to the kit instruction manual. Colorimetric detection and image recording were performed with an ATR03 reader and the IconoClust-AT software, respectively. Positive signal intensities were transformed in a binary code. The binary code corresponding to the core genome was converted to a hexadecimal code as previously described [7].

### Pulsed-field gel electrophoresis (PFGE)

PFGE was performed on 162 isolates of our collection, as previously described [8,31]. In detail, chromosomal DNA was prepared in 2% (wt/vol) low melting point agarose plugs and digested with *Spe*I restriction enzyme at 37°C overnight. Samples were run on 1% (wt/vol) agarose gel in 0.5X TBE buffer at 14°C on a CHEF DR-III PFGE system (Bio-Rad, Hertsfordshire, United Kingdom). PFGE run settings were: initial switching time 5 s; final switching time 45 s; gradient 6 V; run time 21 h. PFGE band patterns were compared as described previously [4] and the PFGE clusters were defined according to the criteria established by Tenover and coworkers [32]. In detail, isolates with band pattern with >85% similarity were refer to as genetically related clones.

### Multilocus sequence typing (MLST)

A total of 80 *P. aeruginosa* independent isolates were typed. MLST was performed as described by Maatallah and co-workers [33]. Briefly, genomic DNA was isolated by using the “DNeasy Blood & Tissue kit” (Qiagen, Valencia, CA, USA) following the manufacturer’s guidelines. DNA amplification of the seven housekeeping genes (*acsA, aroE, guaA, mutL, nuoD, ppsA and trpE*) was performed with a MiniOpticon real-time PCR detection system (Bio-Rad Laboratories, Munich, Germany) using the QuantiTect SYBR Green PCR mix (Qiagen, Valencia, CA, USA). Standard primers [34] were employed as previously described [33]. The specificity of the amplification products was determined by a final melting curve analysis. DNA products were purified and sequenced on both strands by Eurofins MWG Operon GmbH (Ebersberg, Germany) with published primers [33]. Sequences were compared to publicly available MLST databases, accessible on the *P. aeruginosa* MLST website (http://pubmlst.org/paeruginosa). Each isolate was assigned a sequence type (ST) number according to its allelic profile. Genetic distance between MLST profiles was calculated as defined at http://pubmlst.org/analysis/.

### Evaluation of typing methods

The discriminatory index (DI), which indicates the probability for two strains, sampled randomly from a population, to belong to a different type was calculated as previously described [35]. In order to quantify the congruence between typing methods the adjusted Rand coefficient was calculated, using the algorithm available at http://comparingpartitions.info. The first coefficient quantifies the global agreement between two methods, while the second indicates the probability that two strains are coherently classified as the same clone by both methods [35,36].

### Identification of AT cluster of clones

The relatedness between the AT-genotypes was inferred with the eBURST clustering algorithm (http.//eBURST.mlst.net). The algorithm was applied on 13 SNPs located on the core genome plus the *fliCa/b* gene [2]. The eBURST was also employed for comparison to the global *P. aeruginosa* AT-database, derived from 4 previous studies [7,14,15,17].

### Analysis of accessory genome AT-profiles

The distribution of accessory genome markers relative to strain origin or other features was evaluated using the Monte Carlo method [24]. Strains isolated from the same patient with equal profile in the accessory genome were excluded from the analysis.

## Authors’ contributions

AB participated in the design of the study, performed part of the AT assays, performed MLST experiments, analysed AT and MLST data and drafted the manuscript. GS participated in the design of the study, performed part of the PFGE assays, analyzed PFGE data, performed statistical analyses and drafted the manuscript. MK maintained the strain collection and carried out part of the PFGE and AT experiments. OJ conceived the study, participated in its design and coordination and revised the manuscript. NC performed AT-profile evaluation. LW participated to AT-profile evaluation and interpretation, and critically contributed to the revision of the manuscript. All authors read and approved the final manuscript.

## Supplementary Material

Additional file 1 ** Database of the 124*P. aeruginosa*independent isolates within our collection.** The database shows the clinical data of the 124 independent *P.aeruginosa* isolates and presence/absence of accessory genome genes/islands based on microarray typing. On the right, the corresponding AT- and MLST-genotype are provided, as well as the clone cluster ID, according to each of the three genotyping technique employed. ND = not defined; SC = single clone; SP = single pulsotype. Click here for file

Additional file 2 **PFGE dendrogram with assignment of genetically related clones of 162 *P. aeruginosa*isolates of our strain collection.** The UPGMA dendrogram includes a selection of the 124-independent isolates analyzed by microarray typing (in square boxes). The red line represents the 85% similarity value and the square brackets indicate the different clusters identified according to Tenover criteria [32]. Click here for file

Additional file 3 ** Correlation between microarray typing and PFGE typing.** Multi-isolates AT-genotypes are listed in the first column. The distribution of the isolates of each multi-isolate AT-genotype among PFGE pulsotypes is shown in each lane. The frequency data and number of isolates refers exclusively to independent isolates. Click here for file

Additional file 4 **MLST single allele and allelic profile data for all 80 typed isolates.** The database shows for each isolate typed by MLST single allele and allelic profile. Medium-quality allele sequences were not determined (n.d.). Novel allele types and allelic profiles are defined as NEW. The clonal complex corresponding to each ST was added, when available. All data were obtained by comparison to the MLST Public Database (pubmlst.org/paeruginosa). Click here for file

Additional file 5 **Distribution of the 41 AT-genotypes identified among hospital locations.** Venn’s diagram of the AT-genotype distribution among the three hospital locations: Verona, Rovereto, and Trento. Distributions were calculated from the 124 independent *P. aeruginosa* isolates of our collection. (PNG 25 kb) Click here for file

Additional file 6 ** Cluster of AT-clones identified including all available AT-typed*P. aeruginosa*clinical populations.** Cluster of clones were identified by eBurst analysis of our AT-genotypes together with 4 published AT-databases [7,14,15,17]. The colour code indicates the AT-genotypes of our strain collection and for each genotype the% of isolates associated to chronic or acute infections. Novel clones (not described in other studies) are highlighted by a rectangular box. Clones predicted by eBURST as group primary founders are underlined. Click here for file
