# Supplementary figures and images for: Molecular typing and epidemiological investigation of clinical populations of Pseudomonas aeruginosa using an oligonucleotide-microarray
Source: BMC Microbiol. 2012 Jul 27;12:152. doi: 10.1186/1471-2180-12-152 (PMC3431270; doi:10.1186/1471-2180-12-152)

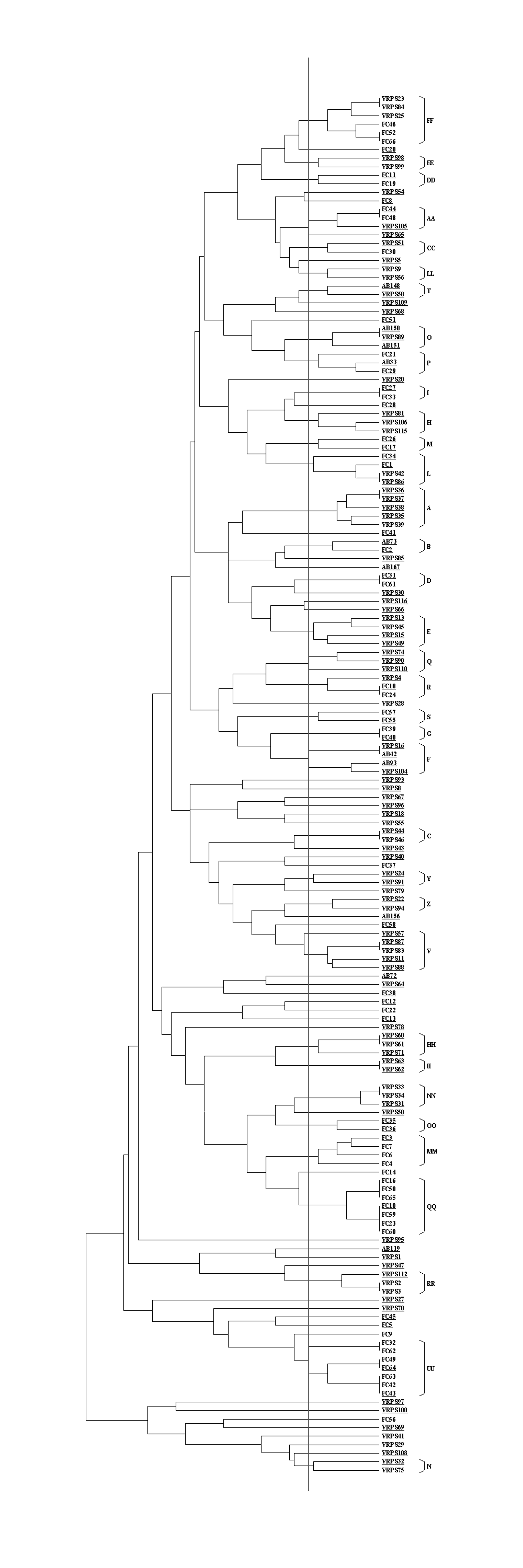

Supplement: Additional file 2 — PFGE dendrogram with assignment of genetically related clones of 162 P. aeruginosaisolates of our strain collection. The UPGMA dendrogram includes a selection of the 124-independent isolates analyzed by microarray typing (in square boxes). The red line represents the 85% similarity value and the square brackets indicate the different clusters identified according to Tenover criteria [32]. [file 1471-2180-12-152-S2.png]

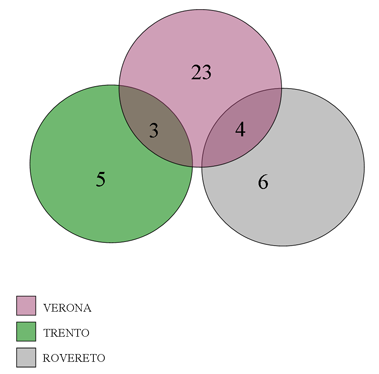

Supplement: Additional file 5 — Distribution of the 41 AT-genotypes identified among hospital locations. Venn’s diagram of the AT-genotype distribution among the three hospital locations: Verona, Rovereto, and Trento. Distributions were calculated from the 124 independent P. aeruginosa isolates of our collection. (PNG 25 kb) [file 1471-2180-12-152-S5.png]

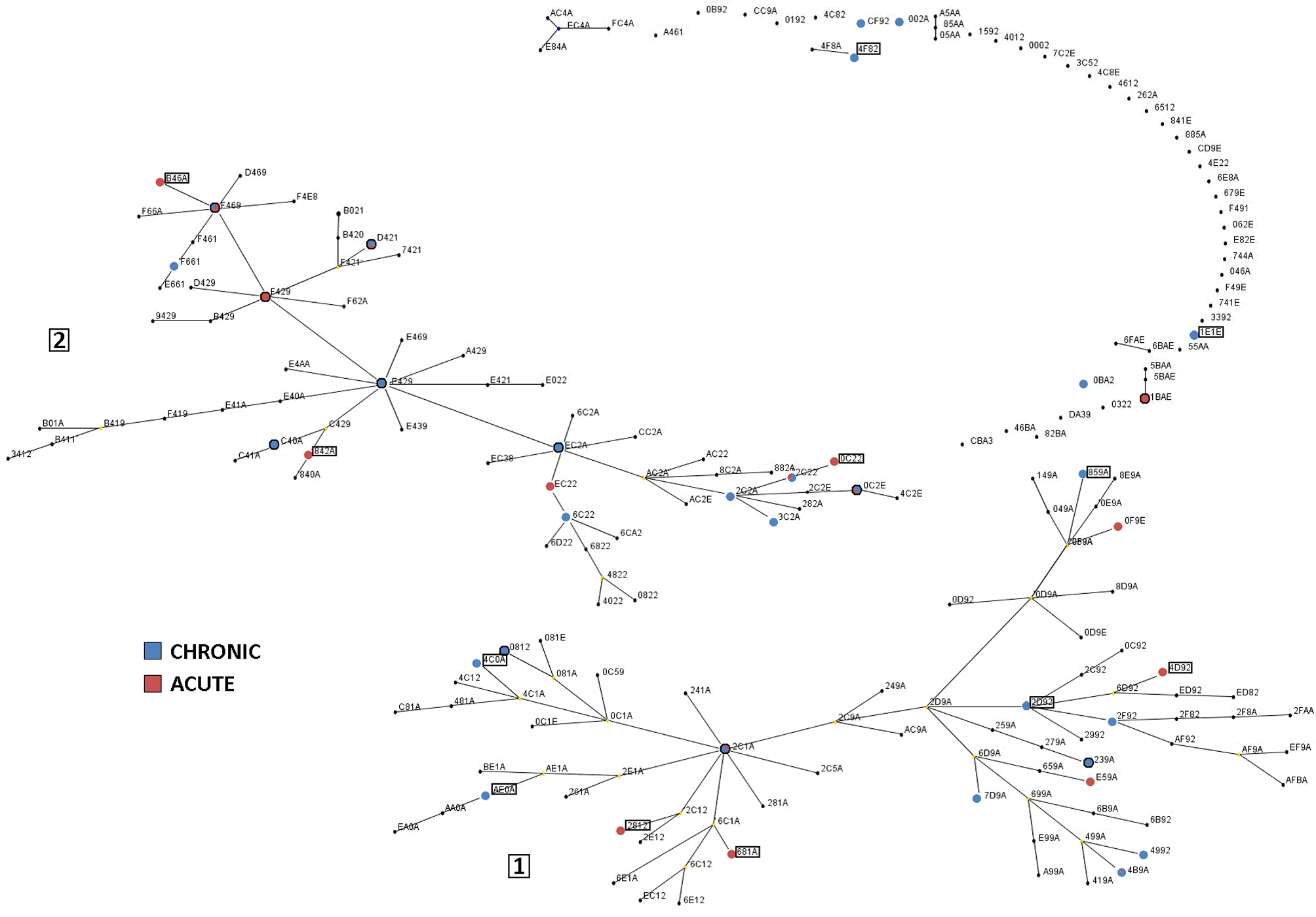

Supplement: Additional file 6 — Cluster of AT-clones identified including all available AT-typedP. aeruginosaclinical populations. Cluster of clones were identified by eBurst analysis of our AT-genotypes together with 4 published AT-databases [7,14,15,17]. The colour code indicates the AT-genotypes of our strain collection and for each genotype the% of isolates associated to chronic or acute infections. Novel clones (not described in other studies) are highlighted by a rectangular box. Clones predicted by eBURST as group primary founders are underlined. [file 1471-2180-12-152-S6.png]
