# Supplementary material for: Molecular typing and epidemiological investigation of clinical populations of Pseudomonas aeruginosa using an oligonucleotide-microarray
Source: BMC Microbiol. 2012 Jul 27;12:152. doi: 10.1186/1471-2180-12-152 (PMC3431270; doi:10.1186/1471-2180-12-152)
Supplement: Additional file 3 — Correlation between microarray typing and PFGE typing. Multi-isolates AT-genotypes are listed in the first column. The distribution of the isolates of each multi-isolate AT-genotype among PFGE pulsotypes is shown in each lane. The frequency data and number of isolates refers exclusively to independent isolates. [file 1471-2180-12-152-S3.doc]

|  |  | **PFGE** | | | | | | | | | | | | | | | | | | | | | | | | | | | | | | | | | | | | |
| --- | --- | --- | --- | --- | --- | --- | --- | --- | --- | --- | --- | --- | --- | --- | --- | --- | --- | --- | --- | --- | --- | --- | --- | --- | --- | --- | --- | --- | --- | --- | --- | --- | --- | --- | --- | --- | --- | --- |
|  | **Clonal groups** | **Total** | **A** | **AA** | **B** | **C** | **CC** | **D** | **DD** | **E** | **EE** | **F** | **G** | **H** | **HH** | **I** | **II** | **L** | **M** | **MM** | **N** | **NN** | **O** | **OO** | **P** | **Q** | **QQ** | **R** | **RR** | **S** | **T** | **U** | **UU** | **V** | **Y** | **Z** | **s.p.** | **n.d.** |
| **AT** | **Total** | 124 | 4 | 2 | 2 | 1 | 1 | 1 | 1 | 3 | 1 | 4 | 1 | 1 | 2 | 1 | 2 | 3 | 2 | 1 | 1 | 1 | 3 | 2 | 2 | 3 | 1 | 2 | 1 | 1 | 2 | 0 | 2 | 4 | 2 | 1 | 44 | 19 |
|  | **1** | 66 | 0 | 0 | 0 | 1 | 1 | 0 | 1 | 3 | 1 | 4 | 1 | 0 | 2 | 1 | 2 | 2 | 2 | 0 | 0 | 0 | 0 | 2 | 1 | 1 | 0 | 0 | 1 | 0 | 0 | 0 | 0 | 3 | 2 | 1 | 23 | 11 |
|  | **2** | 2 | 0 | 0 | 0 | 0 | 0 | 0 | 0 | 0 | 0 | 0 | 0 | 0 | 0 | 0 | 0 | 1 | 0 | 0 | 0 | 0 | 0 | 0 | 0 | 0 | 0 | 0 | 0 | 0 | 0 | 0 | 0 | 0 | 0 | 0 | 0 | 1 |
|  | **3** | 5 | 4 | 0 | 0 | 0 | 0 | 0 | 0 | 0 | 0 | 0 | 0 | 0 | 0 | 0 | 0 | 0 | 0 | 0 | 0 | 0 | 0 | 0 | 0 | 0 | 0 | 0 | 0 | 0 | 0 | 0 | 0 | 0 | 0 | 0 | 0 | 1 |
|  | **4** | 3 | 0 | 2 | 0 | 0 | 0 | 0 | 0 | 0 | 0 | 0 | 0 | 0 | 0 | 0 | 0 | 0 | 0 | 0 | 0 | 0 | 0 | 0 | 0 | 0 | 0 | 0 | 0 | 0 | 1 | 0 | 0 | 0 | 0 | 0 | 0 | 0 |
|  | **5** | 9 | 0 | 0 | 0 | 0 | 0 | 1 | 0 | 0 | 0 | 0 | 0 | 0 | 0 | 0 | 0 | 0 | 0 | 1 | 0 | 0 | 0 | 0 | 0 | 0 | 1 | 0 | 0 | 0 | 0 | 0 | 2 | 0 | 0 | 0 | 4 | 0 |
|  | **6** | 7 | 0 | 0 | 1 | 0 | 0 | 0 | 0 | 0 | 0 | 0 | 0 | 0 | 0 | 0 | 0 | 0 | 0 | 0 | 0 | 0 | 0 | 0 | 0 | 2 | 0 | 0 | 0 | 0 | 1 | 0 | 0 | 0 | 0 | 0 | 3 | 0 |
|  | **7** | 6 | 0 | 0 | 1 | 0 | 0 | 0 | 0 | 0 | 0 | 0 | 0 | 0 | 0 | 0 | 0 | 0 | 0 | 0 | 0 | 0 | 0 | 0 | 0 | 0 | 0 | 1 | 0 | 0 | 0 | 0 | 0 | 0 | 0 | 0 | 4 | 0 |
|  | **8** | 5 | 0 | 0 | 0 | 0 | 0 | 0 | 0 | 0 | 0 | 0 | 0 | 0 | 0 | 0 | 0 | 0 | 0 | 0 | 0 | 0 | 2 | 0 | 1 | 0 | 0 | 0 | 0 | 0 | 0 | 0 | 0 | 0 | 0 | 0 | 1 | 1 |
|  | **9** | 2 | 0 | 0 | 0 | 0 | 0 | 0 | 0 | 0 | 0 | 0 | 0 | 0 | 0 | 0 | 0 | 0 | 0 | 0 | 1 | 1 | 0 | 0 | 0 | 0 | 0 | 0 | 0 | 0 | 0 | 0 | 0 | 0 | 0 | 0 | 0 | 0 |
|  | **10** | 3 | 0 | 0 | 0 | 0 | 0 | 0 | 0 | 0 | 0 | 0 | 0 | 0 | 0 | 0 | 0 | 0 | 0 | 0 | 0 | 0 | 0 | 0 | 0 | 0 | 0 | 0 | 0 | 0 | 0 | 0 | 0 | 0 | 0 | 0 | 2 | 1 |
|  | **11** | 2 | 0 | 0 | 0 | 0 | 0 | 0 | 0 | 0 | 0 | 0 | 0 | 0 | 0 | 0 | 0 | 0 | 0 | 0 | 0 | 0 | 0 | 0 | 0 | 0 | 0 | 0 | 0 | 0 | 0 | 0 | 0 | 0 | 0 | 0 | 2 | 0 |
|  | **rest** | 14 | 0 | 0 | 0 | 0 | 0 | 0 | 0 | 0 | 0 | 0 | 0 | 1 | 0 | 0 | 0 | 0 | 0 | 0 | 0 | 0 | 1 | 0 | 0 | 0 | 0 | 1 | 0 | 1 | 0 | 0 | 0 | 1 | 0 | 0 | 5 | 4 |

**Table S2.** Matrix data file of clonal clusters or complexes identified according to AT and PFGE typing.
